# Genetic association analysis for common variants in the Genetic Analysis Workshop 18 data: a Dirichlet regression approach

**DOI:** 10.1186/1753-6561-8-S1-S70

**Published:** 2014-06-17

**Authors:** Osvaldo Espin-Garcia, Xiaowei Shen, Xin Qiu, Yonathan Brhane, Geoffrey Liu, Wei Xu

**Affiliations:** 1 Department of Biostatistics, Princess Margaret Cancer Centre, 610 University Ave., Toronto, ON, M5G 2M9, Canada; 2 Department of Statistics and Actuarial Science, University of Waterloo, 200 University Avenue West, Waterloo, ON, N2L 3G1, Canada; 3 Samuel Lunenfeld Research Institute, Mount Sinai Hospital, 60 Murray Street, Toronto, ON, M5T 3L9, Canada; 4 Ontario Cancer Institute/Princess Margaret Cancer Centre, 610 University Ave., Toronto, ON, M5G 2M9, Canada; 5 Dalla Lana School of Public Health, University of Toronto, 155 College St., Toronto, ON M5T 3M7, Canada

## Abstract

We propose a genetic association analysis using Dirichlet regression to analyze the Genetic Analysis Workshop 18 data. Clinical variables, arranged in a longitudinal data structure, are employed to fit a multistate transition model in which the transition probabilities are served as a response in the proposed analysis. Furthermore, a gene-based association analysis via penalized regression is implemented using the markers at a single-nucleotide polymorphism level that we previously identified via nonpenalized Dirichlet regression.

## Background

Genetic association analyses have had tremendous successes in recent years; however, most of these analyses were based on binary or continuous responses. Thus we propose a multivariate response vector indicating probabilities of transitions to predefined hypertensive states. This enables us to reflect the inherent uncertainty involved in the probability that a patient will transfer to a given state.

An important feature of our approach is the incorporation of prehypertension as an intermediate state. As Winegarden argues, prehypertension blood pressure in young patients helps predict the development of hypertension [1].

## Methods

### Definition of response

We defined a response summarizing the phenotype information into a vector that will be used in a genetic association analysis. The response is defined as a 3-dimensional vector of probabilities $y=\left(y_{1},y_{2},y_{3}\right),\sum^{y_{j}}=1\left({\text{y}}_{1},{\text{y}}_{2},{\text{y}}_{3}\right),\sum^{{\text{y}}_{\text{j}}}=1$, such that each element measures the probability of a transition to a blood pressure level (normotensive, prehypertensive, or hypertensive) given the previous level.

The analysis was done without the knowledge of the underlying simulation model and we used the real phenotype data only.

### Data quality control

Data quality control was performed in PLINK [2]. We only considered the data from chromosome 3 for analysis. We used a call rate for individuals of 95%, a Hardy-Weinberg disequilibrium test at a significance level of 1 × 10^−6^, and a missing rate of 95% for each marker. Markers with a minor allele frequency of at least 5% were retained for analysis. Additionally, all individuals' time points with at least 1 missing clinical variable were excluded.

### Multistate transition model

We describe hypertension, our trait of interest, using a 3-state model based on recorded blood pressure levels for each individual at each examination. The states are defined as follows: normal blood pressure (state 1) when the systolic blood pressure is less than 120 mm Hg and diastolic blood pressure is less than 80 mm Hg; prehypertension (state 2) when the blood pressure level is not in state 1, the systolic blood pressure is less than 140 mm Hg, and the diastolic blood pressure is less than 90 mm Hg; and hypertension (state 3) for all other cases. Also, if a patient used antihypertensive medication, the state assigned at that examination is hypertension (state 3) regardless of the recorded blood pressure levels. Once the states are defined, we consider a multistate transition model; it is important to note that all 9 transitions are possible.

Our interest in transition models lies in estimating of the transition probabilities as defined in Kalbfleisch and Lawless [3] which are given by

$$P\text{(}S_{i}\left(t\right)=j\;\text{|}\;S_{i}\left(t-1\right)=l,x_{i}\left(t-1\right))=y_{ilj}\left(t\right),l,j\in \left\{1,2,3\right\}$$

where $\left\{S_{i}\left(r\right),r=1,2,\ldots \right\}$ and $\left\{x_{i}\left(r\right)=\left(x_{i1}\left(r\right),\ldots ,x_{ip}\left(r\right)\right),r=1,2,\ldots \right\}$ denote the observed state and the covariates for subject *i *at the $r^{\text{th}}$ examination respectively.

This model takes advantage of the longitudinal data structure and the definition of the response follows naturally. To estimate the transition probabilities, we fit a multinomial regression model, based on covariates (gender, smoking status and age) and the state at the previous examination.

To get expressions for $y_{il}=\left(y_{il1},y_{il2},y_{il3}\right),l=1,2,3,$ we consider a generalized logit model of the form

$$\text{log}\left(y_{ilj}/y_{ill}\right)=z_{il}\gamma_{lj},j=1,2,3,j\neq l$$

besides, $1=y_{ill}+\sum_{j=1,j\neq l}^{3}y_{ilj}=y_{ill}\left(1+\sum_{j=1,j\neq l}^{3}\text{exp}\left(z_{il}\gamma_{lj}\right)\right)$, where $z_{il}=\left(x_{i}\left(l\right),time\right)$ is the observed vector of covariates for subject *i *plus a categorical variable denoting the effect of examination time in the model (and possible interactions), and $\gamma_{lj}$ is the vector of coefficients for the corresponding multinomial regression model.

Thus, a transition probability matrix (TPM) is defined for each individual as follows

$$\text{TP}{\text{M}}_{i}=\left(\begin{array}{ccc} \frac{1}{1+\sum_{j=2}^{3}\text{exp}\left(z_{i1}\gamma_{1j}\right)} & \frac{\text{exp}\left(z_{il}\gamma_{12}\right)}{1+\sum_{j=2}^{3}\text{exp}\left(z_{i1}\gamma_{1j}\right)} & \frac{\text{exp}\left(z_{il}\gamma_{13}\right)}{1+\sum_{j=2}^{3}\text{exp}\left(z_{i1}\gamma_{1j}\right)}\\ \frac{\text{exp}\left(z_{i2}\gamma_{21}\right)}{1+\sum_{j=1,j\neq 2}^{3}\text{exp}\left(z_{i2}\gamma_{2j}\right)} & \frac{1}{1+\sum_{j=1,j\neq 2}^{3}\text{exp}\left(z_{i2}\gamma_{2j}\right)} & \frac{\text{exp}\left(z_{i2}\gamma_{23}\right)}{1+\sum_{j=1,j\neq 2}^{3}\text{exp}\left(z_{i2}\gamma_{2j}\right)}\\ \frac{\text{exp}\left(z_{i3}\gamma_{31}\right)}{1+\sum_{j=1}^{2}\text{exp}\left(z_{i3}\gamma_{3j}\right)} & \frac{\text{exp}\left(z_{i3}\gamma_{32}\right)}{1+\sum_{j=1}^{2}\text{exp}\left(z_{i3}\gamma_{3j}\right)} & \frac{1}{1+\sum_{j=1}^{2}\text{exp}\left(z_{i3}\gamma_{3j}\right)} \end{array}\right)$$

Therefore, the response for subject *i *is a row taken from $\text{TP}{\text{M}}_{i}$ and is determined by conditioning on the patient's last available observed state and covariates.

### Dirichlet regression

Once the response is modeled our objective is to determine whether there is an association between it and the genotypes. We assess this association using Dirichlet regression [4], which suits this response structure. The advantage of this approach lies in its tractability in dealing with the proposed response. It also allows a more comprehensive understanding of the genetic effect on the expression of hypertension, and therefore in its possible interpretation. For instance, if a signal was detected for a marker, it would suggest an association between the marker and the transition of blood pressure states jointly rather than a single level. Therefore, the Dirichlet approach is more informative in the sense of explaining the plausibility of each defined state.

To relate the genetic information and the defined response under a Dirichlet regression approach, the likelihood given each individual's vector of covariates, ${\text{s}}_{i}$, is

$$L=\prod_{i=1}^{n}\left\{\Gamma \left(\Lambda \left({\text{s}}_{i}\right)\right)\overset{3}{\underset{j=1}{\prod }}\frac{y_{ij}^{\lambda_{j}\left(s_{i}\right)-1}}{\Gamma \left(\lambda_{j}\left({textbfs}_{i}\right)\right)}\right\}$$

where $\lambda_{j}\left(s_{i}\right)=\lambda_{ij}>0,\Lambda \left(s_{i}\right)=\Lambda_{i}=\sum_{j=1}^{3}\lambda_{j}\left(s_{i}\right)$ and Γ(⋅) is the gamma function.

The parameters, $\lambda_{j}\left({\text{s}}_{i}\right)$, are defined in terms of a linear predictor using a logarithm link,

$$\text{log}\left(\lambda_{j}\left({\text{s}}_{i}\right)\right)=\text{log}\left(\lambda_{ij}\right)=\sum_{m=1}^{M}\beta_{jm}s_{im}={\text{s}}_{i}\beta_{j}j=1,2,3$$

where *M *is the number of covariates included in the model and $\beta_{j}$ is the vector of regression coefficients that explains the effects (in log scale) of the covariates on the *j*^th ^component.

Considering the above, 2 models are analyzed:

Model 1 (M1): $\text{log}\left(\lambda_{ij}\right)=\alpha_{j}^{M1}+\beta_{j}^{M1}g_{i}^{k}$(base model)

Model 2 (M2):$\text{log}\left(\lambda_{ij}\right)=\alpha_{j}^{M2}+\beta_{j}^{M2}g_{i}^{k}+\text{FA}{\text{M}}_{i}\delta_{j}^{M2}$ (adjusted model)

Here $g_{i}^{k}$ represents the number of copies of the minor allele on the *k*^th ^single-nucleotide polymorphism (SNP) for the *i*^th ^individual under an additive genetic model; $\text{FA}{\text{M}}_{i}$ is the *i*^th ^row of contrast matrix for the pedigree number considered as a categorical variable and $\theta_{j}^{h}={\left(\alpha_{j}^{h},\beta_{j}^{h},{\delta^{h}}_{j}^{t}\right)}^{t}$ is the vector of regression coefficients on the *j*^th ^component. (Note $\delta_{j}^{M1}=0)$.

Our interest in these models lies in the potential genetic effect of each marker on the proposed response. To assess this, Wald statistics were used to test the null hypothesis of no association between each SNP and the response, ${\text{H}}_{0}:\beta =0$ (vs.${\text{H}}_{A}:\text{not}\;{\text{H}}_{0}$), $\beta =\left(\beta_{1},\beta_{2},\beta_{3}\right)$.

### Gene-based association

Once we identify significant SNPs through the genetic association analysis as described above, we proceed to perform the analysis at a gene level. To achieve this, we propose a penalized regression. Including all the markers simultaneously, this penalized method aims to select those SNPs with higher association. The analysis is done on those candidate genes that contain at least 1 significant marker that has already been determined. Variable selection on the SNPs is assessed via a penalized likelihood of the form

$$pl\left(\eta ;Y,G,c,\kappa \right)=l\left(\eta ;Y,G\right)-c\kappa \sum_{l=1}^{p}\sqrt{k}{∥\eta_{\cdot l}∥}_{2}-\left(1-c\right)\kappa \sum_{l=1}^{p}{∥\eta_{\cdot l}∥}_{1}$$

where $l\left(\eta ;Y,G\right)$ represents the log-likelihood of a dirichlet distributed sample with response matrix $Y={\left(y_{1}^{t},\ldots ,y_{n}^{t}\right)}^{t};$$G={\left(g_{1}^{t},\ldots ,g_{n}^{t}\right)}^{t}$ (or G:FAM for **M2**) is the design matrix, $g_{i}=\left(1,g_{i}^{1},\ldots ,g_{i}^{p}\right);$*p *denotes the number of markers considered for variable selection; *k *is the number of states; *η *is the regression coefficients vector; *c *and *λ *are parameters for the penalized regression; and ${∥\eta_{.l}∥}_{2}={\left(\sum_{j=1}^{k}\eta_{lj}^{2}\right)}^{1/2}$ and are the penalty norms. It is important to note that when c=1 we have a ridge regression penalty, whereas when c=0 we have a lasso penalty. We implement the variable selection for the penalized dirichlet regression using R code provided on the Statistical Genetics and Genomics Laboratory at the University of Pennsylvania webpage [5].

## Results

### Data quality results

The Genetic Analysis Workshop 18 (GAW18) data consists of 855 individuals with genotype and phenotype information. As a result of missing data, transition probabilities are estimated for only 835 individuals. Of these, 43 are removed because of low call rate. The overall call rate for the remaining 792 individuals is 99.82%.

The Genome Wide Association Study (GWAS) data includes 65,519 SNPs for chromosome 3, of which 59 are excluded because it is not possible to reliably obtain position information for these markers. The remaining 65,460 SNPs are considered for data quality control. Because of a low genotyping rate, 114 markers are removed; none are excluded by the Hardy-Weinberg equilibrium test; and 13,011 markers are removed because of low minor allele frequency. The remaining 52,357 markers are considered for analysis.

### Analysis results

The parameter estimates for the transition models are obtained using R [6]. To test examination time effect; likelihood ratio tests are performed in which the null model considers only the available clinical variables. Table 1 presents the final transition models.

**Table 1:** Selected transition models

Transition	Model
1→*j*	$\text{log}\left(y_{1j}/y_{11}\right)=\gamma_{1j0}+\left(\gamma_{1j1}x_{sex}+\gamma_{1j2}x_{smoke}+\gamma_{1j3}x_{age}\right)*time\;j=2,3$
2→*j*	$\text{log}\left(y_{2j}/y_{22}\right)=\gamma_{2j0}+\gamma_{2j1}x_{sex}+\gamma_{2j2}x_{smoke}+\gamma_{2j3}x_{age}+time\;j=1,3$
3→*j*	$\text{log}\left(y_{3\text{j}}/y_{33}\right)=\gamma_{3l0}+\gamma_{3j1}x_{sex}+\gamma_{3j2}x_{smoke}+\gamma_{3j3}x_{age}\;j=1,2$

**Figure 1:**
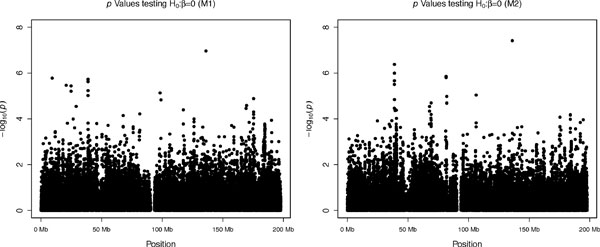
**Manhattan plots for genetic association analysis**.

After the response is estimated, models M1 and M2 are fit using R [7] for each available SNP. Figure 1 displays the Manhattan plots for the *p *values that result from testing the null hypotheses of no association between the markers and the response. The graphs show that only 1 marker under M2 is significant at the standard significance level for GWAS (5 × 10^−8^). Interestingly, the same marker is the most significant marker under M1, although it is not significant at the standard threshold. This suggests that the adjustment for family incorporated in M2 accounts for the family structure in the data. Also, the proposed methodology demonstrates consistency in that the same marker proves to be the most significant under both models. Table 2 summarizes these findings.

**Table 2:** Association analysis results

SNP	Gene	MA	MAF (%)	*p *Value (M1)	*p *Value (M2)
rs12492830	*PCCB*	C	7.22	1.1 × 10^−7^	3.9 × 10^−8^

*MA*, minor allele *MAF*, minor allele frequency.

**Figure 2:**
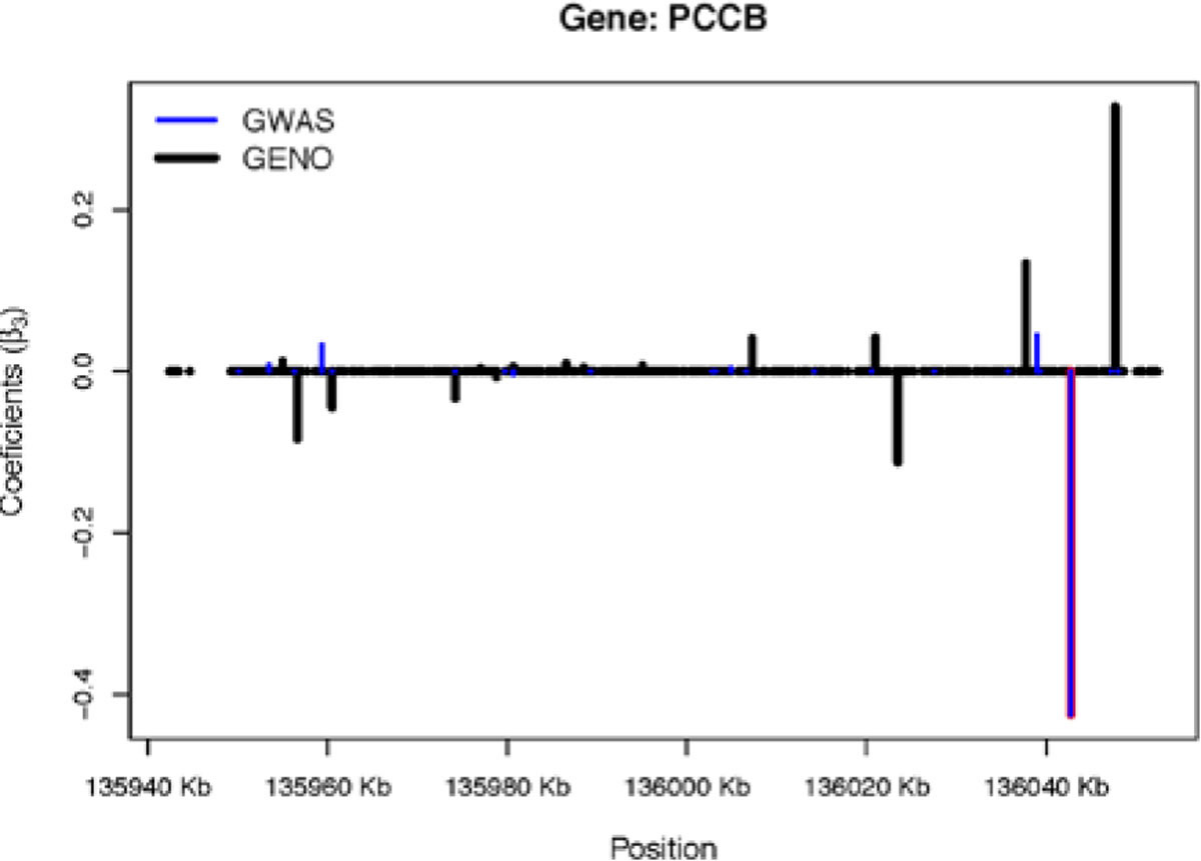
**Penalized regression results for M2 only**.

Once significant markers were identified, a gene-level association analysis is performed using the penalized regression described above for different levels of *c *(0, 0.3, 0.5, 0.7, and 1). The analysis is conducted utilizing both the GWAS and the dosage imputed genotypes (GENO) information as the explanatory variables. Figure 2 shows the penalized regression results for the gene containing the significant SNP (rs12492830) for c=0.5 only. This level of *c *is a blended penalty function, equally weighting the ridge and lasso penalties. Table 3 shows the results for different levels of *c *under M2 for gene *PCCB*.

**Table 3:** Comparison of penalized regression under different levels of *c*

			No. of parameters selected (iterations for convergence)				
**Data**	**# SNPs**	**c=**	**0**	**0.3**	**0.5**	**0.7**	**1**

GWAS	22		10 (260)	10 (148)	8 (135)	7 (163)	7 (174)

GENO	607		42 (427)	35 (199)	24 (176)	25 (136)	19 (115)

## Discussion

The present work implements a multistate transition model that conveniently accommodates the longitudinal data structure. Whether the information contained by the available clinical variables is sufficient for predicting the hypertensive state is debatable, however.

Although the adjusted model (M2) is an improvement over the base model (M1), neither of the described models accounts for correlation between individuals nor heteroscedasticity. One way to possibly overcome this is to incorporate a latent variable into the model. Such an extension follows.

Model 3 (M3):$\text{log}\left(\lambda_{ij}\right)=\alpha_{j}^{M3}+\beta_{j}^{M3}g_{i}^{k}+u_{i}$ where $u_{i}$ is the *i*^th ^element of a vector *u *that follows a MVN(0,K) distribution; here **K **is twice the estimated kinship matrix. In this case, however, the estimation of the parameters of interest, $\beta_{j}$, is not straightforward. Further research of this methodology is warranted.

With respect to the penalized regression, to avoid an arbitrary selection of *c*,a cross-validation method could be implemented.

## Conclusions

We propose a methodology that conveniently uses the longitudinal data structure to define a probabilistic outcome, which, we believe, explains hypertension in a more suitable way. Dirichlet regression provides an interesting approach that, along with other more common responses, can be successfully used in genetic association analysis. Our model finds a statistically significant marker at the standard significant level for GWAS, which is noteworthy, considering that it is often difficult to find association. Moreover, when the penalized method is used on the GENO data we are able to find significant markers in addition to those have already found using GWAS data.

## Competing interests

The authors declare that they have no competing interests.

## Authors' contributions

OEG and WX designed the overall study; OEG conceived the study, conducted statistical analyses, and wrote the manuscript; XS, XQ, and YB helped develop the study; GL revised the clinical aspects of the study. All authors read and approved the final manuscript.

## References

[B1] Winegarden CR From "Prehypertension" to Hypertension? Additional Evidence Ann Epidemiol 2005 15 720 725 10.1016/j.annepidem.2005.02.010 15921930

[B2] Purcell S Neale B Todd-Brown K Thomas L Ferreira MAR Bender D Maller J Sklar P de Bakker PIW Daly MJ PLINK: a toolset for whole-genome association and population-based linkage analysis Am J Hum Genet 2007 81 559 575 10.1086/519795 17701901 PMC1950838

[B3] Kalbfleisch JD Lawless JF The analysis of panel data under a Markov assumption J Am Statist Assoc 1985 80 863 871 10.1080/01621459.1985.10478195

[B4] Campbell G Mosimann JE Multivariate methods for proportional shape ASA Proceedings of the Section on Statistical Graphics 1987 Washington, DC 10 17

[B5] Chen J Li H Variable selection for Dirichlet-multinomial regression for identifying covariates that are associated with microbiomes Ann Appl Stat 2013 7 418 442 10.1214/12-AOAS592 PMC3846354 24312162

[B6] Venables WN Ripley BD Modern Applied Statistics with S 2002 4 New York, Springer

[B7] Maier MJ DirichletReg: Dirichlet Regression in R http://dirichletreg.r-forge.r-project.org Ver. 0.4-0

